# Influence of intercostal muscles contraction on sonographic evaluation of lung sliding: a physiological study on healthy subjects

**DOI:** 10.1186/s44158-024-00168-0

**Published:** 2024-05-06

**Authors:** Daniele Guerino Biasucci, Alessandro Cina, Claudio Sandroni, Umberto Moscato, Mario Dauri, Luigi Vetrugno, Franco Cavaliere

**Affiliations:** 1 Department of Clinical Sciences and Translational Medicine, “Tor Vergata” University of Rome, Rome, Italy; 2 Fondazione Policlinico Universitario “A. Gemelli” IRCCS, Rome, Italy; 3 Catholic University of the Sacred Heart, Rome, Italy; 4 Department of Medical, Oral and Biotechnological Sciences, “G. D’Annunzio” University of Chieti-Pescara, Chieti, Italy

**Keywords:** Lung ultrasound, Critical care ultrasound, Point-of-care ultrasound, Pneumothorax, Intercostal muscles physiology, Respiratory physiology, Respiratory mechanics

## Abstract

**Objectives:**

To investigate the following: (a) effects of intercostal muscle contraction on sonographic assessment of lung sliding and (b) inter-rater and intra-observer agreement on sonographic detection of lung sliding and lung pulse.

**Methods:**

We used Valsalva and Muller maneuvers as experimental models in which closed glottis and clipped nose prevent air from entering the lungs, despite sustained intercostal muscles contraction. Twenty-one healthy volunteers underwent bilateral lung ultrasound during tidal breathing, apnea, hyperventilation, and Muller and Valsalva maneuvers. The same expert recorded 420 B-mode clips and 420 M-mode images, independently evaluated for the presence or absence of lung sliding and lung pulse by three raters unaware of the respiratory activity corresponding to each imaging.

**Results:**

During Muller and Valsalva maneuvers, lung sliding was certainly recognized in up to 73.0% and up to 68.7% of imaging, respectively, with a slight to fair inter-rater agreement for Muller maneuver and slight to moderate for Valsalva. Lung sliding was unrecognized in up to 42.0% of tidal breathing imaging, and up to 12.5% of hyperventilation imaging, with a slight to fair inter-rater agreement for both. During apnea, interpretation errors for sliding were irrelevant and inter-rater agreement moderate to perfect. Even if intra-observer agreement varied among raters and throughout respiratory patterns, we found it to be higher than inter-rater reliability.

**Conclusions:**

Intercostal muscles contraction produces sonographic artifacts that may simulate lung sliding. Clinical studies are needed to confirm this hypothesis. We found slight to moderate inter-rater agreement and globally moderate to almost perfect intra-observer agreement for lung sliding and lung pulse.

**Trial registration:**

ClinicalTrials.gov registration number.

NCT 02386696.

**Supplementary Information:**

The online version contains supplementary material available at 10.1186/s44158-024-00168-0.

## Background

Lung ultrasound (LUS) is more accurate in ruling out a pneumothorax (PNX) than chest X-ray [1–3]. Lung sliding, lung pulse, and B-lines are the three sonographic signs proving that the visceral pleura is in contact with the parietal pleura [1–4]. Lung sliding originates from the movement of the visceral pleura over the parietal pleura during tidal ventilation, being visualized with ultrasound as a sort of “to-and-fro” movement of the pleural line [5–7]. Lung pulse reflects changes in heart volume during the cardiac cycle transmitted to the pleural surface [1, 3, 7]. B-lines are vertical artifacts perpendicular to the pleural line, whose semiquantitative assessment reflects water/gas ratio [1, 7]. The presence of lung sliding and lung pulse plays a major role in excluding PNX because, in the absence of interstitial or alveolar lung diseases, B-lines are not commonly seen on the anterior surface of the chest, where partial or occult PNX can be detected in the supine patient. Lung sliding and lung pulse are commonly visualized using the brightness mode (B-mode). The presence of lung sliding can also be confirmed by the seashore sign or excluded by the stratosphere sign using the motion mode (M-mode) [4, 6].

In critical care settings, the specificity of absent lung sliding in ruling out PNX ranges from 78% up to 98%, with a negative predictive value close to 100% [4, 8–10]. However, false-positive cases may occur when the visceral pleura is in contact with the parietal pleura, but it does not slide because of the following: (a) no volume of air enters the respiratory system (e.g., apnea or respiratory arrest), one single lung (as in the case of inadvertent bronchial intubation or main bronchus obstruction), and a segment of the lung (e.g., atelectasis); (b) lung or pleural diseases, such as ARDS or pleural adhesions; and (c) dynamic hyperinflation from airway obstruction (e.g., asthma) [4, 11]. The sensitivity of absent lung sliding in ruling out PNX ranges from 81% up to 91% [4, 8–10]. A particular category of false-negative cases has been described in recent years. In 2014, Cavaliere and coworkers published a case series of eight postoperative patients in ICU in whom LUS showed artifacts mimicking lung sliding on the side of pneumonectomy, despite the complete absence of the lung and, therefore, of the visceral pleura [12]. The mimicked lung sliding appeared only during spontaneous respiration after successful weaning from mechanical ventilation, and it was not detectable, while the patients were sedated, paralyzed, and on mechanical ventilation. Further cases of spontaneously breathing patients with a confirmed diagnosis of PNX on computed tomography and a simultaneous presence of lung sliding on LUS have been reported [13–15]. A possible explanation of this phenomenon might be that chest muscles contraction during spontaneous breathing makes detection of lung sliding difficult. In fact, during inspiration, the parasternal intercostal muscles move ventrally and straighten, while internal intercostal muscles have a prevalent expiratory activity; therefore, the intercostal space width varies through the respiratory cycle [16, 17]. Since the parietal pleura covers the inner surface of the thoracic wall, being separated by intercostal muscles only by the endothoracic fascia, we hypothesized that LUS might detect a false movement of the parietal pleura induced by intercostal muscles contraction occurring in severe dyspnea.

In the light of this evidence, this study was aimed at investigating sonographic effects of the sustained contraction of intercostal muscles. The secondary aim was to assess inter-rater reliability and intra-observer agreement of sonographic detection of lung sliding and lung pulse.

## Methods

### Design, participants, and settings

This prospective observational study was performed on a cohort of 21 healthy volunteers recruited among physicians, nurses, and other allied healthcare personnel at the Policlinico “A. Gemelli” University Hospital in Rome. Subjects with present or past clinical history of respiratory or cardiovascular diseases were not considered for inclusion.

The study was approved by the Ethics Committee of the Catholic University of the Sacred in Rome: approval number 1436/15. All enrolled subjects gave their informed consent following Ethics Committee recommendations. The study was registered on ClinicalTrials.gov (NCT 02386696).

### General protocol

The participants were asked to perform five different respiratory procedures: three respiratory maneuvers (Apnea, Valsalva, and Mueller) and two different respiratory patterns (tidal ventilation during quiet breathing and hyperventilation), as described below. Mueller and Valsalva maneuvers were considered as a physiological model to investigate effects of the sustained contraction of intercostal muscles, since during these conditions no lung volume changes are allowed because of a closed mouth and clipped nose. Before starting the study, all subjects were appropriately trained to perform the required respiratory procedures by one of the authors (D. G. B.).

Ultrasound examination was performed on both sides during each phase by an expert critical care physician proficient in critical care ultrasound (D. G. B.), and all imaging was stored.

### Respiratory maneuvers

**Fig. 1:**
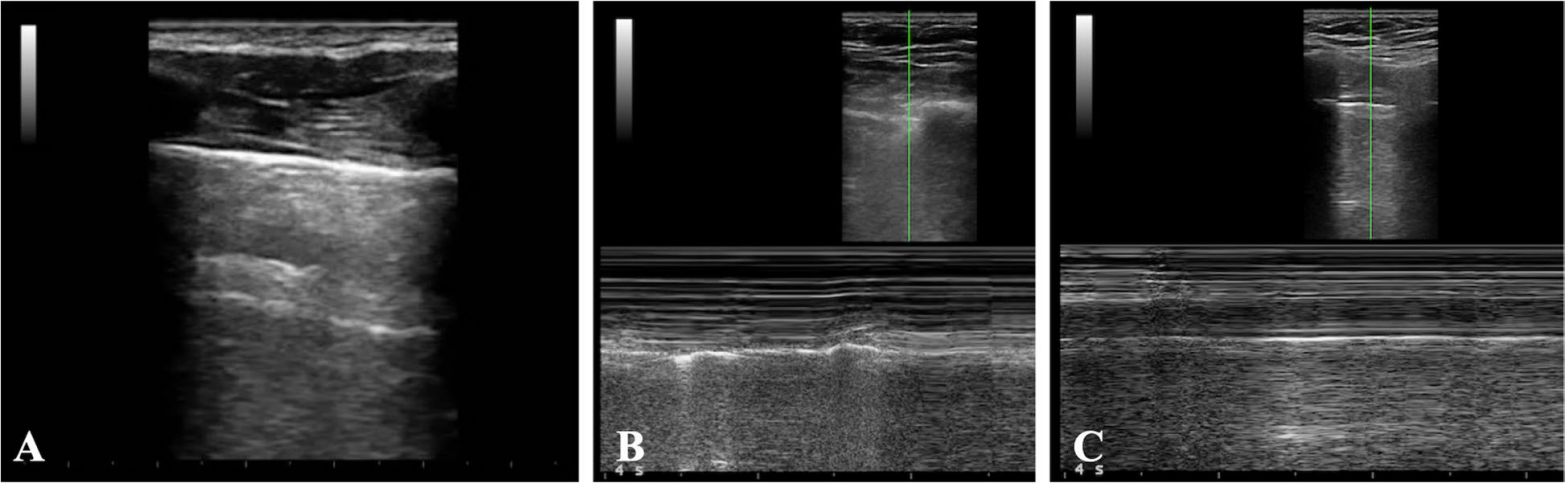
False lung sliding during Muller and Valsalva maneuvers. **A** Placing the probe perpendicular to two consecutive ribs in the parasternal area allowing visualization of the so-called “bat sign”: the upper and lower ribs are the wings of the bat, and, a little deeper, the pleural line is the body of the bat. **B** *False lung sliding during Muller’s maneuver*. M-mode imaging reproducing artifacts mimicking lung sliding which is generated by contractions of parasternal intercostal muscles during Muller’s maneuver. **C** *False lung sliding during Valsalva’s maneuver*. M-mode imaging reproducing artifacts mimicking lung sliding which is generated by contractions of parasternal intercostal muscles during the Valsalva maneuver

- *Tidal ventilation* at quiet breathing: Initially, the volunteers were asked to breathe quietly to allow the operator to identify the “bat sign” and achieve the correct angle of insonation (Fig. 1A).
- *Apnea*: Subjects were asked to hold their breath for 10 s while keeping the intercostal muscles relaxed and inhibiting diaphragmatic activity.
- *Mueller maneuver* (Fig. 1B): Subjects were asked to expire forcibly through the mouth, after exhalation of normal tidal volume, to residual volume and to maintain it with a clipped nose. As soon as ultrasound imaging was stabilized, the volunteers performed a sustained maximal inspiratory effort while their mouth closed and nose clipped (Mueller maneuver).
- *Valsalva maneuver* (Fig. 1C): Subjects were asked to inspire rapidly close to total lung capacity and maintain this volume actively for 2–3 s; then, they performed a sustained maximal expiratory effort while their mouth closed and nose clipped (Valsalva maneuver).
- *Hyperventilation*: Subjects were asked to take deep and rapid breaths to simulate polypnea.

After each maneuver and before performing the next one, the subjects were allowed to recover by breathing quietly for 2 min at least. The sequence was performed twice on both sides of the chest wall.

### Ultrasound assessment and acquisition

LUS was performed while the subjects were seated on a chair with a back support to minimize trunk motion and enable the operator to maintain a steady insonation angle during the imaging acquisition.

The MyLab Five Esaote Ultrasound System (Esaote SpA, Genoa, Italy) was used, equipped with a high frequency 4–13 MHz broadband linear probe, well suited for sonographic examination of pleural line and parasternal intercostal muscles.

Imaging acquisition was obtained by placing the probe longitudinally along the midclavicular line and perpendicular to two consecutive ribs, at the level of the 3rd to the 4th intercostal spaces. This scan offers a good view of the ribs, intercostal muscles, and pleural lines (Fig. 1A). When the correct position of the probe with proper image acquisition was achieved, the operator registered and saved a 10-s video clip in B-mode for each side and two sequences of six consecutive breaths in M-mode during each respiratory maneuver. Videotapes and images were all stored on a memory disc by the principal investigator (D. G. B.).

### Ultrasound interpretation and bias assessment

The same researcher (D. G. B.) who performed ultrasound examinations renamed videos and images saved based on a random sequence, making it impossible to trace the corresponding maneuver from the file name. The process was repeated twice so that two groups of files, A and B, were finally obtained, in which the same imaging was stored under different names and sequences.

Three researcher experts in critical care ultrasound (A. C., C. S., F. C.), who were blinded to the file encoding system, examined group A first and group B 15 days later. During each evaluation session, first, each rater was asked to assess whether lung sliding and lung pulse were present in each B-mode video, integrating it with the corresponding M-mode imaging and then without interacting with other investigators. The access to the related M-mode imaging was allowed only after that the three raters had sent the final assessment based on B-mode to the principal investigator (D. G. B.). Then, the assessment based on the integration of B-mode videos and M-mode images was registered and analyzed separately from the assessment based solely on B-mode. Before starting the study, the three examiners agreed on the criteria for lung sliding and lung pulse presence after two meetings.

### Statistical analysis

The Shapiro–Wilk test assessed the normal distribution of continuous variables, which is most powerful for small sample sizes with less than 50 patients.

Continuous variables with normal distribution are presented as mean ± standard deviation (SD). Continuous variables with non-normal distribution are presented as the median and interquartile range (IQR). Categorical variables were presented as the number of patients, or percentages, with 95% CIs and were compared using the *χ*^2^ test.

Inter-rater and intra-observer agreement was assessed using Cohen’s k test, comparing observed agreement with the expected agreement if the ratings were independent. Cohen’s kappa ranges between − 1 and 1. According to Landis and Koch, values < 0 indicate no agreement, values between 0 and 0.20 slight agreement, values between 0.21 and 0.40 fair agreement, values between 0.41 and 0.60 moderate agreement, values between 0.61 and 0.80 substantial agreement, and values > 0.81 almost perfect agreement [18].

No statistical sample size calculation was performed a priori since this is a physiologic study on healthy volunteers with no previously published trial testing the same hypothesis.

STATA software for Mac (Stata/BE 17 for Mac, StataCorp., 4905 Lakeway College Station, USA) was used. *P* < 0.05 was considered statistically significant.

## Results

Subjects enrolled were all males, aged 38 (± 11) years, and with a body mass index (BMI) of 24.9 (± 2.4) kg/m^2^. During the study, 420 B-mode clips and M-mode images were recorded, stored, and examined.

The main results are summarized in Tables 1, 2 and 3 and in Supplemental Tables 1 and 2.

**Table 1:** Error rate in detecting lung sliding and lung pulse based on the integration of B-mode and M-mode imaging across three different respiratory patterns and two respiratory maneuvers

	**Rater 1 vs expected**			**Rater 2 vs expected**			**Rater 3 vs expected**		
	Agreement % (95% CI)	Uncertain % (95% CI)	Error % (95% CI)	Agreement % (95% CI)	Uncertain % (95% CI)	Error % (95% CI)	Agreement % (95% CI)	Uncertain % (95% CI)	Error % (95% CI)
**Lung sliding**									
**Tidal breathing**	58.3 (43.7–61.2)	14.6 (7–28)	27.1 (16.2–41.6)	6.2 (2.0–17.7)	54.2 (40.0–67.6)	42 (28.6–56)	70.1	27.1 (16.4–41.3)	2.1 (0.3–13.5)
**Apnea**	100	0	0	85.4 (72.3–92.9)	12.5 (5.7–25.2)	2.1 (0.3–13.4)	100	0	0
**Muller**	23 (13–37)	4 (1.0–15.3)	73 (58.7–83.6)	31.2 (19.7–45.6)	56.2 (42.0–69.5)	14.5 (7.1–27.6)	54.2 (40.0–67.6)	25 (14.7–39.1)	20.8 (11.5–34.6)
**Valsalva**	27.1 (16.4–41.3)	4.2 (1.1–15.3)	68.7 (54.4–80.2)	64.6 (50.1–76.8)	31.2 (19.7–45.6)	4.2 (1.0–15.2)	60.4 (46.0–73.2)	27.1 (16.4–41.3)	14.6 (7.1–27.6)
**Hyperventilation**	100	0	0	52.1 (38.1–65.7)	35.4 (23.2–49.8)	12.5 (5.7–25.2)	68.7 (54.4–80.2)	27.1 (16.4–41.3)	4.2 (1.0–15.3)
**Lung pulse**									
**Tidal breathing**	91.7 (79.7–96.8)	0	8.3 (3.1–20.3)	81.2 (67.6–89.9)	0	18.8 (10.0–32.3)	70.8 (56.5–82.0)	0	29.2 (18.0–43.5)
**Apnea**	100	0	0	97.9 (86.5–99.7)	0	2.1 (0.3–13.4)	93.7 (82.2–98.0)	0	6.2 (2.0–17.7)
**Muller**	50 (36.1–63.8)	0	50 (36.1–63.8)	83.3 (70.0–91.4)	0	16.7 (8.5–30.0)	31.3 (19.7–46.6)	0	68.7 (54.4–80.2)
**Valsalva**	39.5 (26.8–36.9)	0	60.5 (46.0–73.2)	87.5 (75.0–94.3)	0	12.5 (5.7–25.2)	25 (14.7–39.1)	0	75 (61.0–85.2)
**Hyperventilation**	4.2 (1.0–15.5)	0	95.8 (84.7–98.9)	43.7 (30.5–58.0)	0	56.3 (42.0–69.5)	0	0	100

Agreement, agreement between observed and expected for each rater; uncertain, unable to assess (%); error, error rate; hyperpnea, hyperventilation

**Table 2:** Inter-rater agreement for the assessment of lung sliding and lung pulse based on the integration of B-mode and M-mode imaging

	Cohen’s *k*		
	**Rater 1 vs 2**	**Rater 2 vs 3**	**Rater 1 vs 3**
**Lung sliding**			
**Tidal breathing**	0.12	0.11	0.12
**Apnea**	0.46	0.41	1
**Muller**	0.10	0.25	0.23
**Valsalva**	0.13	0.10	0.10
**Hyperventilation**	0	0.31	0.10
**Lung pulse**			
**Tidal breathing**	0.54	0.16	0.12
**Apnea**	0.48	0.40	0.23
**Muller**	0.10	0	0.35
**Valsalva**	0.10	0.10	0.61
**Hyperventilation**	0.14	0	0

**Table 3:** Intra-rater agreement for the assessment of lung sliding and lung pulse based on the integration of B-mode and M-mode imaging

	Cohen’s *k*		
	**Rater 1**	**Rater 2**	**Rater 3**
**Lung sliding**			
**Tidal breathing**	0.41	0.93	0.85
**Apnea**	0	1	0
**Muller**	0.47	0.85	0.55
**Valsalva**	0.46	0.96	0.53
**Hyperventilation**	0	1	0.58
**Lung pulse**			
**Tidal breathing**	0.10	1	0.81
**Apnea**	0	1	0.54
**Muller**	0.58	1	0.65
**Valsalva**	0.61	1	0.83
**Hyperventilation**	0.25	1	0

Table 1 reports the agreement between what each rater observed and what was expected, across two different respiratory maneuvers and three different respiratory patterns. The error rate in detecting lung sliding and lung pulse has been also reported in Table 1.

Table 2 reports inter-rater agreement of the joint evaluation of B-mode and M-mode imaging for lung sliding and lung pulse.

Table 3 reports the intra-observer reliability of the joint assessment of B-mode and M-mode imaging for lung sliding and lung pulse.

Supplemental tables report inter-rater (eTable 1) and intra-observer (eTable2) agreement for lung sliding and lung pulse, based solely on the evaluation of B-mode imaging. No differences were found between the two hemithoraces.

### Tidal breathing

During tidal breathing, the raters failed to recognize lung sliding in up to 42.0% of imaging and lung pulse in up to 29.2% (Table 1). Based on the integration of B-mode and M-mode imaging, inter-rater agreement was slight for lung sliding and slight to moderate for lung pulse (Cohen’s *k* 0.54 for rater 1 vs 2; Cohen’s *k* < 0.20 for rater 1 vs 3 and rater 2 vs 3; Table 2). Cohen’s *k* pointed out perfect or almost perfect intra-observer agreement for raters 2 and 3, for both lung sliding and lung pulse; on the other hand, only moderate intra-observer reliability for lung sliding and slight for lung pulse were found for rater 1 (Table 3). Based on B-mode imaging only, inter-rater agreement was worse for both lung sliding and lung pulse (eTable 1), while intra-observer agreement remained substantially unchanged (eTable 2).

### Apnea

During apnea, inter-rater agreement was moderate for lung pulse (Table 2), while inter-rater agreement for lung sliding was moderate to perfect (Cohen’s *k* 1 for rater 1 vs 3; Cohen’s *k* 0.41 and 0.46 for rater 2 vs 3 and rater 1 vs 2, respectively; Table 2), based on the integration of B-mode and M-mode. Cohen’s *k* pointed out a very poor intra-observer agreement for raters 1 and 3 in detecting lung sliding and a perfect intra-observer agreement for rater 2 (Table 3). For lung pulse, a moderate to perfect intra-observer agreement was found for raters 2 and 3 and no agreement for rater 1 (Table 3). Based solely on B-mode imaging, inter-rater agreement was found to be worse while intra-observer agreement to be improved (supplemental tables).

### Muller maneuver

During Muller maneuvers, lung sliding was certainly recognized in 14.9 up to 73.0% of imaging and uncertain in up to 56.2% (Table 1). Inter-rater agreement was slight to fair for both lung sliding and lung pulse (Table 2). Cohen’s *k* pointed out a moderate to almost perfect or perfect intra-observer agreement, for both lung sliding and lung pulse detection, all based on the integration of B-mode and M-mode imaging (Table 3).

### Valsalva maneuver

During Valsalva, lung sliding was certainly recognized in up to 68.7% of imaging and uncertain in up to 31.2% (Table 1). Inter-rater agreement was slight for lung sliding and slight to moderate for lung pulse (Table 2). Cohen’s *k* pointed out a moderate to almost perfect intra-observer agreement for lung sliding and a substantial to perfect intra-observer agreement for lung pulse detection (Table 3).

### Hyperventilation

During hyperventilation, lung sliding was erroneously unrecognized in up to 12.5% of imaging and judged uncertain in 27.1 to 35.4% of imaging by two out of three raters (Table 1). All raters failed to correctly recognize lung pulse in most imaging obtained during hyperventilation (Table 1). Inter-rater agreement was slight to fair for lung sliding and very poor for lung pulse (Table 2). Cohen’s *k* pointed out a wide range of intra-observer agreement among raters, from poor to perfect, both for lung sliding and lung pulse (Table 3).

Based solely on the evaluation of B-mode imaging obtained during Muller and Valsalva maneuvers and hyperventilation trials, inter-rater reliability, as well as intra-observer agreement, remained globally and substantially similar when compared to those based on the integration of B-mode and M-mode imaging, both for lung sliding and lung pulse (supplemental tables).

## Discussion

Data from the present study showed that the three raters reported lung sliding in most sonographic imaging obtained during Valsalva and Muller maneuvers, in which glottis and nose closure prevented the air from entering the lungs (Table 1). For this reason, during Valsalva and Muller maneuvers, the visceral pleura does not slide over the parietal pleura, as no air enters the lungs. Therefore, sonographic lung sliding should not be visualized in a healthy subject performing Muller or Valsalva maneuvers. The prolonged and maximal contraction of respiratory muscles performed during the maneuvers may explain these findings. The presence of artifacts mimicking lung sliding and produced by the contraction of parasternal intercostal muscles has been previously described in a case series of eight ICU patients after pneumonectomy. In this case series, artifacts were present during spontaneous breathing but were absent during mechanical ventilation under apneic sedation [12, 14]. Similar artifacts may occur in patients suffering from dyspnea [13–15]. Both recently pneumonectomized patients and dyspneic patients may share an increased activity of the parasternal intercostal muscles, whose contraction stabilizes the rib cage and contributes to inspiration by moving against a pleural pressure gradient in opposition to the deflationary action of the diaphragm [16, 17, 19]. Our findings allow to clarify the mechanisms originating these artifacts. In fact, by varying the width of intercostal spaces, the active contraction of intercostal muscles during Muller and Valsalva maneuvers may make the parietal pleura move falsely by dragging. This phenomenon may have significant implications for LUS examination in clinical practice. In fact, in patients recruiting intercostal accessory muscles due to respiratory distress, artifacts mimicking lung sliding (Fig. 1B–C) may erroneously lead clinicians to rule out an eventual PNX.

In our study, artifacts induced by the maximal muscular activity performed during Valsalva and Muller maneuvers, as during hyperventilation trials, may have hidden lung pulse in most videos and images (Fig. 1B–C). Finally, the recognition of the sonographic lung sliding may be insufficient to exclude an eventual PNX if substantial intercostal muscle activation is detected on clinical examination or by ultrasound. However, these findings should be interpreted cautiously since Cohen’s *k* pointed out only a slight to fair inter-rater agreement for Muller and Valsalva maneuvers.

The secondary endpoint of this study was to assess inter-rater and intra-observer agreement on the sonographic assessment of lung sliding and lung pulse. In this regard, three conditions were studied first. During apnea, the air does not enter the lungs, so we expected to find the lung pulse but not the lung sliding. During tidal breathing and hyperventilation, lung pulse and lung sliding were expected to be present. The three raters correctly identified lung sliding during hyperventilation in most imaging but missed it in almost one-third of cases during tidal breathing. Lung sliding was erroneously recognized in very few imaging obtained during apnea. Furthermore, lung pulse was correctly recognized in almost the total of imaging during apnea and tidal breathing, but it was almost totally missed during hyperventilation. A possible explanation of missing lung pulse during tidal ventilation is that the sign is more or less apparent in the parasternal area depending on the tidal volume and the respiratory pattern, whether predominantly thoracic or abdominal, which may depend on age, gender, and position [17]. On the other hand, lung pulse was probably missed in most hyperventilation imaging because hidden by more profound and more prolonged lung sliding. A remarkable finding was the slight to fair inter-rater agreement for lung sliding detection and slight to moderate for lung pulse, except for apnea condition in which inter-rater agreement resulted much better. In this regard, it is essential to consider that all the raters were proficient in critical care ultrasound, and that their performances were comparable, with more than 5 years of experience each in performing LUS on a daily basis. Thus, a possible explanation of these findings represents, at the same time, a limitation of this study whose design differed significantly from clinical practice. In fact, in the latter, LUS is integrated with a clinical examination so that, for instance, a spontaneously breathing patient can be invited to breathe deeper to magnify lung sliding. Furthermore, the operator who performs LUS examination can achieve unlimited imaging by changing the probe position and insonation angle in case of doubt and comparing B-mode and M-mode in each position. Conversely, in this study, the raters judged based on post-processed clips and images. However, to the best of our knowledge, our findings align with data from a recently published clinical study in which a low agreement was found in the interpretation of LUS for PNX diagnosing on critically ill patients [20].

The main limitation of the present study is represented by the fact that even if Valsalva and Muller’s maneuvers have been carefully performed in the way to minimize pressure changes that could have accounted for recruitment of alveolar units due to volume shifts, it was not possible to completely rule out eventual minimal volume changes that may have been caused by the variable degree of alveolar compression, while subjects maintained the maximal muscular contraction. Therefore, these findings need to be confirmed by larger clinical trials.

However, PNX diagnosis consists in a thoughtful reasoning which requires following a specific algorithm including different ultrasound artifacts to be integrated to the clinical presentation and its evolution [1, 4]. Furthermore, a better understanding of sonographic lung sliding amplitude and their determinants is needed for more accurate diagnosing and monitoring of the critically ill.

## Conclusions

This study suggests that the contraction of intercostal muscles may produce sonographic artifacts mimicking lung sliding. However, clinical studies are needed to confirm this finding.

Finally, based on the integrated evaluation of B-mode and M-mode imaging, our study found a slight to moderate inter-rater agreement and a globally moderate to almost perfect intra-observer agreement for lung sliding and lung pulse detection.

### Supplementary Information

Supplementary Material 1: Electronic Supplementary Tables. eTable 1. Inter-rater agreement for lung sliding and lung pulse based solely on B-mode imaging. eTable 2. Intra-rater agreement for lung sliding and lung pulse based solely on B-mode imaging.

## Data Availability

No datasets were generated or analysed during the current study.

## Abbreviations

LUS: Lung ultrasound
PNX: Pneumothorax
B-mode: Brightness mode
M-mode: Motion mode
SD: Standard deviation
IQR: Inter-quartile range
BMI: Body mass index
