# Supplementary material for: Influence of intercostal muscles contraction on sonographic evaluation of lung sliding: a physiological study on healthy subjects
Source: J Anesth Analg Crit Care. 2024 May 6;4:31. doi: 10.1186/s44158-024-00168-0 (PMC11075244; doi:10.1186/s44158-024-00168-0)
Supplement: Supplementary file 1 — Supplementary Material 1: Electronic Supplementary Tables. eTable 1. Inter-rater agreement for lung sliding and lung pulse based solely on B-mode imaging. eTable 2. Intra-rater agreement for lung sliding and lung pulse based solely on B-mode imaging. [file 44158_2024_168_MOESM1_ESM.docx]

**eTable 1.** Inter-rater agreement for lung sliding and lung pulse based solely on B-mode imaging.

| Lung Sliding | Cohen’s *k* | | |
| --- | --- | --- | --- |
|  | **Rater 1 vs 2** | **Rater 2 vs 3** | **Rater 1 vs 3** |
| **Tidal Breathing** | 0 | 0.10 | 0.12 |
| **Apnea** | 0.21 | 0.25 | 1 |
| **Muller** | 0 | 0.21 | 0.11 |
| **Valsalva** | 0.10 | 0.23 | 0.24 |
| **Hyperventilation** | 0.14 | 0.27 | 0.10 |
| Lung Pulse |  |  |  |
| **Tidal Breathing** | 0 | 0.10 | 0.10 |
| **Apnea** | 0 | 0 | 0 |
| **Muller** | 0 | 0 | 0.29 |
| **Valsalva** | 0.14 | 0.10 | 0.54 |
| **Hyperventilation** | 0 | 0 | 0 |

**eTable 2.** Intra-rater agreement for lung sliding and lung pulse based solely on B-mode imaging.

| Lung Sliding | Cohen’s *k* | | |
| --- | --- | --- | --- |
|  | **Rater 1** | **Rater 2** | **Rater 3** |
| **Tidal Breathing** | 0.55 | 0.86 | 0,67 |
| **Apnea** | 1 | 0.96 | 1 |
| **Muller** | 0.36 | 0.92 | 0,71 |
| **Valsalva** | 0.46 | 0.96 | 0,68 |
| **Hyperventilation** | 0.17 | 0.88 | 0,64 |
| Lung Pulse |  |  |  |
| **Tidal Breathing** | 0.64 | 1 | 0,59 |
| **Apnea** | 1 | 1 | 0,36 |
| **Muller** | 0.34 | 1 | 0,62 |
| **Valsalva** | 0.68 | 1 | 0,88 |
| **Hyperventilation** | 0 | 1 | 0.65 |
